# Examining the impact of economic abuse on survivors of intimate partner violence: a scoping review

**DOI:** 10.1186/s12889-022-13297-4

**Published:** 2022-05-19

**Authors:** Laura Johnson, Yafan Chen, Amanda Stylianou, Alexandra Arnold

**Affiliations:** 1 Temple University, 1301 Cecil B. Moore Avenue #543, Philadelphia, PA 19122 USA; 2 Rutgers, The State University of New Jersey, Piscataway, NJ USA; 3 Easterseals New Jersey, East Brunswick, NJ USA; 4 Columbia University, New York, NY USA

**Keywords:** Economic abuse, Financial abuse, Intimate partner violence, Domestic violence, Scoping review

## Abstract

**Background:**

Economic abuse is a unique form of intimate partner violence (IPV) and includes behaviors that control a survivor’s ability to acquire, use, and maintain resources. These tactics can result in someone becoming economically dependent on their partner and may limit their ability to leave the relationship and establish independence. The aim of this study was to conduct a scoping review focused on the impact of economic abuse on survivors of IPV.

**Methods:**

A total of 14 databases were reviewed, which resulted in 35 peer-reviewed manuscripts for inclusion in the study. Manuscripts were included if they were: written in English, published since the year 2000, focused specifically on the impact of economic abuse perpetrated by an intimate partner, economic abuse was measured as an independent variable, and if economic abuse was looked at separately from other forms of IPV. Both convenience and population-based samples were included in the review. Information was extracted using a data charting form. The data were analyzed using a combination of grouping techniques and constant comparison methods to identify key findings.

**Results:**

Studies found significant associations between economic abuse and a range of outcomes, such as mental and physical health, financial impacts, parent-child interactions, and quality of life. The most frequently examined were mental health, followed by financial issues.

**Conclusions:**

Limitations of these studies included a lack of longitudinal research and a focus on heterosexual relationships with male-perpetrated violence toward female survivors. Study findings highlight the wide-ranging potential impacts of economic abuse on survivors and the need for additional research to better understand potential outcomes and implement and evaluate interventions to address them.

## Introduction

Domestic violence, also known as intimate partner violence (IPV), is a serious public health concern that affects countless people each year. The Centers for Disease Control and Prevention (CDC) defines IPV as “physical violence, sexual violence, stalking, or psychological harm by a current or former partner or spouse” [1]. Physical, sexual, and other non-physical forms of abuse such as psychological and emotional abuse behaviors have long been identified as forms of IPV. Only more recently has economic abuse, as its own unique form of abuse, been more deliberately researched. Economic abuse encompasses behaviors that control a survivor’s “ability to acquire, use, and maintain resources thus threatening [their] economic security and potential for self-sufficiency” [2]. Among service seeking samples, approximately 76 to 99% of survivors report experiencing economic abuse [2–5].

Stylianou et al. provided evidence that there are six unique forms of abuse. These include psychological abuse, physical violence, sexual violence, economic control, employment sabotage, and economic exploitation [2]. Economic exploitation occurs when someone intentionally destroys or depletes a survivor’s financial resources or credit [5]. Economic exploitation encompasses behaviors like stealing from an intimate partner, gambling of joint money, opening credit lines without a survivor’s permission, or refusing to pay bills with the intent to ruin a survivor’s credit [6–9]. Economic control involves preventing survivors from having knowledge or access to bank accounts, credit cards, and other shared assets. It can also include denying a survivor access to food, clothing, or medications and tracking a survivor’s use of money [6, 9, 10]. Employment sabotage includes behaviors that prevent a survivor from obtaining or maintaining employment [2], such as forbidding or interfering with a survivor’s employment or education, harassing a survivor at their place of work, and interfering with a survivor obtaining other forms of income including disability and child support [9, 11].

There are some spatial dynamics that make economic abuse unique compared to other forms of abuse [5]. Afrouz highlights ways in which technology has “transcend[ed] communications beyond physical limits,” which has had significant implications for survivors of IPV [12]. While physical abuse requires close proximity to a survivor, technology has enabled abusive partners to implement a range of other control tactics without physical contact. For example, economic abuse can be engaged in from anywhere, with little to no contact with the survivor. This makes it increasingly difficult to end economic abuse, even post separation when the abusive partner no longer has physical access to the survivor [5, 6]. Further, a survivor may not realize that their abusive partner is engaging in these behaviors until significant debt or credit damage has ensued.

Attention to economic abuse is critical, as economic stability is a social determinant of health that significantly influences the physical and mental health and safety of IPV survivors. Economic abuse can have devastating long-term effects on quality of life, financial security, and independence. For example, many perpetrators of IPV use the consumer credit industry to destroy their partners’ financial credit situation [7]. Not only does this cause financial strain, but it also makes it difficult for survivors to leave their relationships when they are ready to do so. Within the United States credit scores are evaluated when individuals are applying for housing, utilities, employment, and insurance [7, 13, 14]. Therefore, credit damage caused by economic abuse tactics such as coerced debt may limit the economic resources and opportunities available to survivors, keeping them entrapped in the abusive relationship and at continued risk for violence.

As the body of literature available on economic abuse has grown, the pervasiveness of economic abuse and its impacts has become increasingly more evident. While a few studies have reviewed the literature on economic abuse broadly, to the authors’ knowledge no studies have conducted a scoping review focused on the impact of economic abuse. Given the uniqueness of economic abuse and its impact on long-term financial capabilities, it is critical that the field intentionally focuses on better understanding the nature and consequences of this type of abuse. The aim of this study is to conduct a scoping review of peer-reviewed literature focused on the impact of economic abuse on survivors of IPV and identify current gaps in research.

## Methods

The decision was made to conduct a scoping review of the literature, as the aim of the study was to methodologically identify and examine the available literature focused on the impact of economic abuse [15]. Study procedures were guided by Arksey and O’Malley’s methodological framework for conducting scoping reviews, which includes identifying a research question, identifying relevant studies, selecting studies for inclusion, charting the data, and summarizing and reporting findings [15]. The PRISMA-ScR Checklist guided the reporting of study methods and findings [16]. While an a priori review protocol was developed, the protocol was not registered. The research question guiding the study was: What is known from the existing literature about the impacts of economic abuse on survivors of IPV?

### Search strategy

A comprehensive search of the literature was conducted of 14 main databases across the fields of Social Work, Sociology, Psychology, Public Health, Women’s and Gender Studies, Criminal Justice, and Economics. Databases searched included: Social Service Abstracts, ProQuest Social Science Collection, Sociological Abstracts, APA PsychInfo, Medline, PubMed, Web of Science, Criminal Justice Abstracts, and Applied Social Sciences Index and Abstracts. The initial search was conducted in April 2021 and updated in March 2022. Search terms used included violence keywords (“intimate partner violence” OR “intimate partner abuse” OR “domestic violence” OR “domestic abuse” OR “dating violence” OR “battered women”) AND economic abuse keywords (“economic abuse” OR “financial abuse” OR “coerced debt” OR “economic control” OR “employment sabotage” OR “economic exploitation” OR “financial exploitation”). The same search strategy was used for all databases.

### Eligibility criteria

To identify studies that focused on the impact of economic abuse on survivors of IPV, the following inclusion criteria were used: (a) full-text publications written in the English language, (b) published in the year 2000 or later in a peer-reviewed journal, (c) the focus of the article was specifically on the impact of economic abuse perpetrated by an intimate partner, (d) economic abuse was measured as an independent variable, and (e) economic abuse was looked at separately from IPV (i.e., measures of IPV that included economic abuse items but that did not separate them out as part of analysis were excluded). Studies with both convenience and population-based samples were included in the review. The decision was made to include studies from 2000 or later because the term “economic abuse” was rarely used in the literature before that time [17]. Literature written in English was selected given the costs associated with translation [15].

Data management was facilitated through Covidence, a cloud-based platform that can be used to organize, screen, and analyze documents for systematic reviews. One member of the research team conducted the initial search. Search results were uploaded into EndNote, a citation management software, and then transferred to Covidence once the search was complete.

The initial search for articles was conducted in April 2021 and resulted in 3472 articles. In March 2022, an updated search was conducted using five primary databases (i.e., Social Service Abstracts, ProQuest Social Science Collection, Medline, PubMed, Criminal Justice Abstracts) to include articles published in 2020 and 2021 that may have been missed during the first search; 187 articles were identified. The authors also reviewed the reference lists of three review articles [6, 17, 18] for additional publications for possible inclusion; six were identified. As such, a total of 3665 were imported for screening. Covidence removed 2325 articles due to duplication. A total of 1340 manuscripts were screened for eligibility for inclusion based on title and abstract. A study met the criteria for inclusion if it was focused on economic abuse as a form of IPV and reported on its impact. A total of 1060 articles were excluded based on this criteria. In the next phase of screening, the full text for 280 articles were assessed; 232 were excluded due to not meeting inclusion criteria. The most common reasons why articles were excluded were that the articles did not mention economic abuse (*n* = 120) or the study did mention economic abuse but was not focused on the impact of this form of IPV on survivors (*n* = 77). Four members of the research team assisted with screening; each manuscript was screened by two individuals. In instances where the screeners were in disagreement about whether a manuscript met inclusion criteria, a third member of the research team reviewed and resolved the discrepancy.

**Fig. 1:**
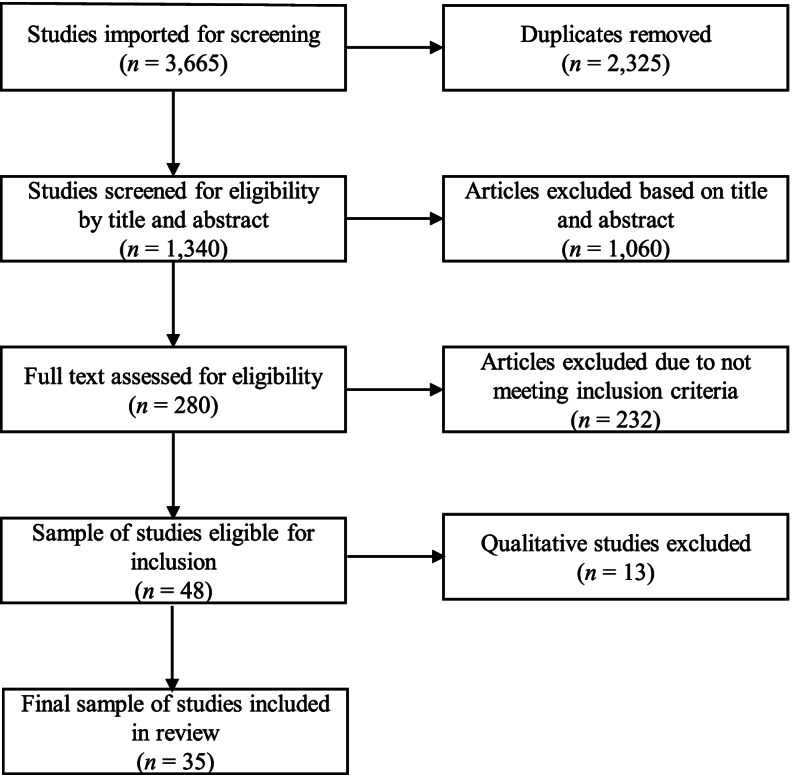
PRISMA flow chart of study selection process for inclusion in scoping review

By the end of the screening process, 48 studies were eligible for inclusion. The focus of this review was on quantitative research; 13 qualitative studies were removed from the sample. A total of 35 studies were identified for inclusion in this review. The PRISMA figure summarizing the review process is illustrated in Fig. 1.

### Data extraction and analysis

Data extraction and analysis was guided by Rodgers and colleagues’ methodological guidance on the synthesis of study findings [19], in addition to Arksey and O’Malley’s scoping review methodology [15]. As a first step, general information about the study was extracted using a data charting form. The form was used to document: (a) sample demographics, (b) research questions, aims, or hypotheses, (c) study methods, (d) how economic abuse was defined and measured, (e) how outcome variables were measured, (f) study findings, (g) study strengths and limitations, and (h) recommendations for future research. Using a tabular format, the research team documented descriptive information about the studies such as sample size, country of origin, and measures used. The textual descriptions were then reviewed closely to extract more detailed information about study methods, findings, limitations, and recommendations. To ensure rigor, a second member of the research team reviewed the data extracted for accuracy. The data were analyzed using a combination of grouping techniques and constant comparison methods to identify key findings [19]. To organize study findings, the studies were grouped by the outcome they focused on, which resulted in six groups. Because this is a scoping review, no critical appraisal tool was utilized. All studies were weighted equally in the presentation of study findings, regardless of rigor [15].

## Results

A total of 35 peer-reviewed manuscripts were included in this review. Table 1 presents the descriptive characteristics of these studies.

**Table 1:** Study characteristics and measurement of economic abuse

Author (Publication year)	Study location	Sample	Nature of study	Measurement of economic abuse	Economic abuse prevalence rate
Adams & Beeble (2019) [20]	United States	Women receiving services from DV and SA service agencies (*n* = 94)	Survey data collected as part of a larger, longitudinal evaluation of an advocacy intervention	SEA (28 items)	Not reported
Adams et al. (2008) [2]	United States	Women receiving services from DV service agencies (*n* = 103)	Cross-sectional survey focused on validating a measurement tool for EA	SEA (28 items)	99% (since relationship began)
Adams et al. (2015) [3]	United States	Women receiving services from DV and SA service agencies (*n* = 93)	Survey data collected as part of a larger, longitudinal evaluation of an advocacy intervention	SEA (28 items)	All reported some form of EA at baseline (since relationship began)
Adams et al. (2020) [21]	United States	Women receiving services from DV service agencies (*n* = 248)	Cross-sectional survey focused on validating a measurement tool for EA	SEA2 (14 items)	96% (at least one EA tactic since relationship began)
Adams et al. (2020) [22]	United States	Women who called the National DV Hotline (*n* = 1823)	Cross-sectional convenience sample using brief surveys	Three (3) items measuring coerced debt	52% (lifetime coerced debt)
Antai et al. (2014) [23]	Philippines	Women between the ages of 15–49 living in Philippines (*n* = 9316)	Cross-sectional representative sample using surveys	Four (4) items measuring EA	Not reported
Bulut et al. (2017) [24]	Turkey	Postpartum women receiving care in a family practice clinic (*n* = 128)	Cross-sectional convenience sample using surveys	Not indicated	3% (timeframe unclear)
Cardenas et al. (2021) [25]	United States	Latina women receiving services from DV agencies (*n* = 200)	Survey data collected as part of a larger, longitudinal evaluation of a financial empowerment program	SEA-12 (12 items)	Not reported
Davila et al. (2021) [26]	United States	Latina women receiving services from DV agencies (*n* = 245)	Cross-sectional study using data collected from a longitudinal evaluation of a financial empowerment program	SEA-12 (12 items)	Not reported
Gibbs et al. (2018) [27]	South Africa	Women aged 18–30 living in informal settlements (*n* = 680)	Cross-sectional study using data collected from a longitudinal evaluation of a DV intervention	Four (4) items measuring EA	52% (at least one EA tactic in past 12 months)
Gottlieb & Mahabir (2021) [28]	United States	Mothers interviewed in hospitals after giving birth (*n* = 3515)	Secondary analysis of longitudinal data from the FFCWB Study	Two (2) items measuring financial control and work/school sabotage	One-third of sample (since the birth of their child)
Gul et al. (2020) [29]	Turkey	Mothers of children referred for pediatric health services (*n* = 336)	Cross-sectional convenience sample using surveys	One (1) item measuring EA	12.5% (since relationship began)
Gurkan et al. (2020) [30]	Turkey	Pregnant women presenting to the antenatal polyclinic (*n* = 370)	Cross-sectional convenience sample using surveys	One (1) item from DV Against Women Screening Form	25.9% (during pregnancy)
Haj-Yahia (2000) [31]	Palestine	Married Palestinian women (*n* = 1334)	Cross-sectional systematic random sample using surveys	Two (2) items measuring financial control	44% (past 12 months)
Hamdan-Mansour et al. (2011) [32]	Jordan	Ever married women over the age of 18 living in villages in southern Jordan (*n* = 807)	Cross-sectional study using stratified random sampling to survey participants	Marital Abuse Scale (5 items)	Not reported
Huang et al. (2013) [33]	United States	Mothers interviewed in hospitals following giving birth (*n* = 2107)	Secondary analysis of longitudinal data collected from the FFCWB Study	Two (2) items measuring financial control and work/school sabotage	11.8% at baseline; 13.5% at Year 3; 15.1% at Year 5 (past 12 months)
Huang et al. (2015) [34]	United States	Mothers interviewed in hospitals following giving birth (*n* = 2410)	Secondary analysis of longitudinal data collected from the FFCWB Study	Two (2) items measuring financial control and work/school sabotage	28% (when their child was one or three years old)
Jewkes et al. (2003) [35]	South Africa	Women between the ages of 18–49 living in South Africa (*n* = 1164)	Cross-sectional representative sample using surveys	Items measuring financial control (number of items unclear)	Not reported
Kanougiya et al. (2021) [36]	India	Ever-married women between ages 18–49 living in two informal settlements (*n* = 4906)	Cross-sectional systematic random sample	15 items measuring EA	23% (at least one form over their lifetime)
Kapiga et al. (2017) [37]	Tanzania	Ever partnered women participating in microfinance loan groups (*n* = 1021)	Cross-sectional baseline survey from a cluster RCT	WHO Violence Against Women Instrument (3 items)	34% (past 12 months)
Johnson (2021) [38]	United States	Pregnant women in a relationship (*n* = 183)	Cross-sectional convenience sample using surveys recruited via research panel service	SEA2 (14 items)	Not reported
Nicholson et al. (2018) [39]	United States	Mothers interviewed in hospitals birth (*n* = 2389)	Secondary analysis of longitudinal data collected from the FFCWB Study	Two (2) items measuring financial control and work/school sabotage	28% (lifetime at Year 1 and Year 3)
Postmus et al. (2012) [40]	United States	Mothers interviewed in hospitals following giving birth (*n* = 2305)	Secondary analysis of longitudinal data collected from the FFCWB Study	Two (2) items measuring financial control and work/school sabotage	Not reported
Postmus et al. (2012) [4]	United States	Women receiving services from DV programs (*n* = 120)	Cross-sectional study using data collected from a longitudinal evaluation of a financial empowerment program	SEA (28 items)	94.2% (in current relationship or last 12 months of most recent relationship)
Postmus et al. (2021) [41]	Cambodia, China, Papua New Guinea, Sri Lanka	Women between the ages of 18–49 (*n* = 3105)	Cross-sectional study using multi-stage cluster sampling to survey participants	Four (4) items measuring EA	35.6% (lifetime)
Sauber et al. (2020) [42]	United States	Female DV survivors recruited through agencies providing services to survivors, as well as online (*n* = 147)	Cross-sectional convenience sample using surveys	SEA-12 (12 items)	95% (at least one experience in the past 6 months)
Stockl & Penhale (2015) [43]	Germany	Women between the ages of 16–86 who received a letter inviting them to participate (*n* = 10,264)	Secondary analysis of cross-sectional nationally representative data collected as part of the Health, Well-Being and Personal Safety of Women in Germany study	Items measuring financial control (number of items unclear)	12% of participants 16–49; 14% 50–65; 13% 66–86 (occurred with current partner)
Stylianou (2018) [44]	United States	Women receiving services from DV agencies (*n* = 457)	Cross-sectional study using data collected from a longitudinal evaluation of a financial empowerment program	SEA-12 (12 items)	93% (past 12 months)
Tenkorang & Owusu (2019) [45]	Ghana	Ever-married women aged 18 and older living within selected communities (*n* = 2289)	Cross-sectional study using multi-stage simple random sampling to survey participants	Seven (7) items measuring employment sabotage, economic exploitation, and economic depravation	8.5% employment sabotage; 24% economic exploitation; 42% economic deprivation (timeframe unclear)
Usta et al. (2007) [46]	Lebanon	Women seeking services in selected health clinics (*n* = 1415)	Cross-sectional convenience sample using surveys	One (1) item measuring EA	12% (lifetime)
Voth Schrag (2015) [47]	United States	Mothers interviewed in hospitals following giving birth (*n* = 2775)	Secondary analysis of longitudinal data collected from the FFCWB Study	Two (2) items measuring financial control and work/school sabotage	14% (timeframe unclear)
Voth Schrag et al. (2019) [48]	United States	Women enrolled in community college (*n* = 435)	Cross-sectional study using simple random sample to survey participants	SEA-12 (12 items)	Not reported
Voth Schrag et al. (2020) [49]	United States	Women enrolled in community college (*n* = 435)	Cross-sectional study using simple random sample to survey participants	SEA-12 (12 items)	43.8% (at least one form of EA in past 12 months)
Yau et al. (2020) [50]	Hong Kong	Adults between the ages of 35–60 (*n* = 504)	Cross-sectional stratified systematic sample using surveys	Chinese SEA-12 (C-SEA-12; 12 items)	36.5% (past 12 months)
Yunus et al. (2017) [51]	Malaysia	Adults aged 60 or older living within selected districts (*n* = 1927)	Longitudinal study using multi-stage cluster sampling strategy and administrative records	Adapted version of the Conflict Tactics Scale for Elder Abuse	8.1% (experienced since turning age 60)

*DV* Domestic violence, *SA* Sexual assault, *EA* Economic abuse, *PTSD* Post-traumatic stress disorder, *FFCWB* Fragile Families and Child Well-Being Study, *SEA* Scale of Economic Abuse, *RCT* Randomized controlled trial, *WHO* World Health Organization

### Study characteristics

Over half of the studies (*n* = 19) in this scoping review collected data from samples within the United States [2–4, 20–22, 25, 26, 28, 33, 34, 38–40, 42, 44, 47–49]. Three articles came from Turkey [23, 24, 27], two came from South Africa [29, 30] and one article came from Germany [31], Ghana [32], Hong Kong [35], India [36], Jordan [37], Lebanon [41], Malaysia [43], Palestine [45], Philippines [46], and Tanzania [50]. A single article used data collected as part of a multi-country study of China, Cambodia, Papua New Guinea, and Sri Lanka [51].

Across the 35 studies, 26 unique datasets were used. Six studies used data from the Fragile Families and Child Wellbeing Study [28, 33, 34, 39, 40, 47]. Adams and Beeble [20] and Adams et al. [3] looked at the same sample that was derived from a larger, longitudinal study evaluating a community-based advocacy intervention. Similarly, Davila et al. [26], Stylianou [44], and Cardenas et al. [25] used data collected as part of larger, longitudinal evaluation of the *Moving Ahead* financial empowerment program. Two studies by Voth Schrag et al. looked at data collected from a sample of women attending a community college [48, 49].

Approximately three-fourths of studies (*n* = 25) utilized cross-sectional designs [2, 4, 21–24, 26, 27, 29–32, 35–38, 41–46, 48–50]. Three studies looked at data with five time points [3, 20, 25], three had four time points [28, 33, 34], three had three time points [39, 40, 47], and one had two time points [51].

For approximately one-third of studies, participants were recruited from domestic violence organizations (*n* = 9); in one study participants were recruited from a domestic violence hotline [22]. Participants were also frequently recruited from their households (*n* = 10) and maternal health clinics or hospitals where participants had recently given birth (*n* = 8).

### Sample

The sample size across studies ranged from 93 to 10,264 participants. All but two studies [50, 51] had entirely female samples. The race/ethnicity of the sample was not documented in 16 studies [23, 24, 27, 29–32, 36, 37, 41, 43, 45–47, 50, 51]. Seven of the studies reported having a sample in which 50% or more identified as white [2–4, 20, 21, 22]. In 30% of the studies (*n* = 8) no one group had 50% or more of any one ethnicity in their sample [25, 26, 28, 33, 34, 38]. Two studies in the United States had entirely Latina samples [39, 40]. For almost all of the studies, the sexual orientation of the participant and/or the gender of their abuser was unclear. However, many used masculine pronouns in survey items (e.g., “he tried to prevent you from going to work/and or school” [42]), suggesting that these studies may have focused on opposite-sex relationships. Only one study clearly indicated that the abusers were all male [42] and two studies clearly indicated that the sample included individuals in both same and opposite-sex relationships [48, 49].

### Defining and measuring economic abuse

Economic abuse was not defined in seven of the studies. Although definitions of economic abuse were generally similar across the 23 studies that included them, there was some variation in the specific language used. Studies described economic abuse as a mechanism of coercive control [2, 3, 20–22, 26, 36, 42], an attitude or behavior [45], or an abusive behavior [47]. These strategies hinder a woman’s ability to acquire, use, and maintain economic resources [2–4, 20–23, 44], threatening her economic security [2, 3, 20–23, 36, 41, 44, 45], economic self-sufficiency [2–4, 17, 20, 21, 23, 26, 36, 38], and increasing financial dependence on their abusive partner [39, 41, 44, 45, 48]. Some studies described economic abuse in terms of the three constructs identified in theoretical and measurement literature [49]: economic control (*n* = 10), employment sabotage (*n* = 7), and economic exploitation (*n* = 4).

The most commonly used measure of economic abuse used across studies was the Scale of Economic Abuse (SEA) or one of its variations [2–4, 20]. The Scale of Economic Abuse is a 28-item measure of economic abuse that includes two subscales – economic control and economic exploitation [2]. Postmus et al. reduced the SEA from 28 items to 12 and identified a three-factor solution that included economic control, economic exploitation, and also employment sabotage [52]. This measure, named the SEA-12 was used in six of the studies [25, 26, 42, 44, 48, 49]. In addition, the SEA-12 was adapted for use in China; the Chinese SEA-12 was used in one study [50].

In 2020, Adams et al. revised the original SEA because the authors felt that the original scale did not adequately measure economic abuse as a form of coercive control and insufficiently addressed the role of the consumer credit system as part of economic abuse. This revised, 14-item scale was named the SEA2 and was used in two studies [21, 38].

Other scales used to measure economic abuse across studies included the Domestic Violence Against Women Screening Form (DVAWS) [53], used in one study [30]; a measure of domestic violence developed by Haj-Yahia [54] for use with Arab survivors [31, 32] or an adaptation [46]; and an adapted version of the Conflict Tactics Scale for elder abuse [55] used in one study [51]. The studies that analyzed the Fragile Families and Child Wellbeing Study data measured economic abuse using two items: “He tried to prevent you from going to work and/or school” and “He withheld money, made you ask for money, or took your money” [27, 28, 33, 34, 37, 39]. Two studies used measures from the United Nations Multi-Country Study, which included four economic abuse tactics: preventing women from earning money, taking her money, throwing her out of the home, or spending money on alcohol, tobacco, or himself when it was needed for the household [40, 41]. One study used the World Health Organization Violence Against Women Instrument, which included three economic abuse items translated into Swahili [47].

The remaining studies either did not use a validated scale [22, 23, 28, 29, 36, 43, 45] or did not indicate how economic abuse was measured [24].

### Outcomes and covariates

#### Outcomes

Study outcomes are presented in Table 2 and can be organized into six categories: (a) financial outcomes (e.g., financial resources, material hardship), (b) mental health (e.g., depression, anxiety), (c) physical health (e.g., mortality, pregnancy symptoms), (d) parenting and child-related outcomes (e.g., use of spanking, engagement in parent-child activities), and (f) quality of life, and (g) other (e.g., mothers’ future criminal justice involvement and union formation).

**Table 2:** Study outcomes of interest and key findings

Author (Publication year)	Type of analysis	Outcomes of interest	Measurement of outcome	Key finding(s)
Adams & Beeble (2019) [20]	Multivariate	Quality of life	9-item Quality of Life Scale adapted for use with survivors [56]	Within-woman change in EA was negatively associated with change in quality of life over time
Adams et al. (2008) [2]	Multivariate	Economic hardship	13-item Economic Hardship Index developed for study	EA was positively associated with economic hardship
Adams et al. (2015) [3]	Multivariate	Perceived financial resources	Adequacy of Financial Support Scale [57]	EA was negatively associated with baseline financial resources; Within-woman change in EA over time was negatively associated with change in financial resources
Adams et al. (2020) [21]	Multivariate	Material dependency	One (1) item asking to what extent survivor relies on the financial resources of their partner	Economic restriction was positively associated with material dependence; EA was positively associated with outstanding debt
		Outstanding debt	6-item index asking participants about what they currently owed money on (e.g., student loan)	
Adams et al. (2020) [22]	Multivariate	Credit damage	One (1) item asking if credit report or credit score has been hurt by the actions of their partner	Coerced debt significantly predicted credit damage and financial dependence
		Financial dependence	One (1) item asking if individual stayed in relationship longer than they wanted to due to financial concerns	
Antai et al. (2014) [23]	Multivariate	Psychological distress	One (1) item asking whether the individual experienced mental health symptoms (e.g., depression) as a result of husband’s acts	Two of four EA items associated with greater odds of suicide attempt; Two of the EA items were associated with greater odds of psychological distress; One EA item associated with lower odds of psychological distress
		Suicide attempt	One (1) item asking if individual ever attempted suicide as a result of husband’s acts	
Bulut et al. (2017) [24]	Bivariate	Postpartum depression	10-item Edinburgh Postpartum Depression Scale [58]	No significant differences in postpartum depression among women exposed to EA compared to those who were not
Cardenas et al. (2021) [25]	Multivariate	Quality of life	9-item Quality of Life Scale adapted for use with survivors [56]	Economic control was significantly and negatively associated with quality of life; however, relationship was no longer significant after controlling for economic empowerment indicators
Davila et al. (2021) [26]	Multivariate	Depression	20-item Center for Epidemiologic Studies Depressed Mood Scale (CES-D) [59]	EA did not lead to a significant increase in R^2^ for depression, anxiety, and PTSD
		Anxiety	7-item Generalized Anxiety Disorder-7 (GAD-7) [60]	
		PTSD	9-items adapted from National Comorbidity Survey [61]	
Gibbs et al. (2018) [27]	Bivariate	Depression	20-item Center for Epidemiologic Studies Depressed Mood Scale (CES-D) [59]	Experiencing any EA was significantly associated with increased depression scores; experienced two or more forms of EA was significantly associated with suicidal ideation
		Suicidal ideation	One (1) item asking if individual has thought about ending their life in the past 4 weeks	
Gottlieb & Mahabir (2021) [28]	Multivariate	Mother’s criminal justice involvement	One (1) item that asks individual if they had been charged with a crime or booked by police for anything other than a minor traffic violation in the past 4 years	Odds of experiencing criminal justice involvement were higher for mothers experiencing EA
Gul et al. (2020) [29]	Bivariate	Contentment with life	5-item Contentment with Life Scale [62]	EA was not significantly associated with contentment with life nor physical or emotional abuse toward child
		Physical or emotional abuse toward child	Two (2) items asking individuals if they had applied physical violence or emotional violence to their child when they are angry with their husbands due to their behaviors	
Gurkan et al. (2020) [30]	Bivariate	Pregnancy symptoms	41-item Pregnancy Symptoms Inventory (PSI) [63]	Fatigue and mental health symptom scores were higher for women experiencing EA
Haj-Yahia (2000) [31]	Multivariate	Self-esteem	Adapted version of Index of Self Esteem (ISE) [64]	The more EA experienced the lower their self-esteem and higher their anxiety and depression
		Anxiety	Adapted version of Costello-Comrey Depression and Anxiety Scale [65]	
		Depression		
Hamdan-Mansour et al. (2011) [32]	Bivariate	Psychological wellbeing	18-item Psychological Well-Being Scale Short Form [66]	Two of six domains of psychological wellbeing (self-acceptance and environmental mastery) were significantly negatively associated with EA
Huang et al. (2013) [33]	Multivariate	Union formation	Multiple items (e.g., marital status, cohabitation status) were used to create four mutually exclusive relationship categories	EA at Year 1 was associated with lower odds of being married or cohabiting at Year 5
Huang et al. (2015) [34]	Multivariate	Early delinquency	Sum of 17 delinquent acts (e.g., run away from home) from the “Things that you have done” scale modeled after the National Youth Survey [67]	Experiencing EA was positively associated with child delinquency at 9 years old, as well as negatively associated with parental involvement
		Parental involvement	Average of individual’s engagement in eight parenting activities (e.g., reading stories)	
		Child neglect	10-item Parent-Child Conflict Tactics Scale [68]	
		Physical punishment		
Jewkes et al. (2003) [35]	Multivariate	Discussion of HIV in relationship	Questions on whether the individuals had ever discussed HIV with their partner and whether they had suggested condom use to their partners	Suggesting condom use in the past year was positively associated with financial abuse
		Condom use		
Kanougiya et al. (2021) [36]	Multivariate	Depression	Patient Health Questtionaire-9 (PHQ-9) [69]	Women who experienced EA had higher odds of experiencing depression, anxiety, and suicidal ideation
		Anxiety	Generalized Anxiety Disorder-7 (GAD-7) [60]	
		Suicidal ideation	Two (2) items asking whether the individual had considered attempting suicide or ever attempted suicide in the past 12 months	
Kapiga et al. (2017) [37]	Multivariate	Psychological distress symptoms	Self-Reporting Questionnaire-20 (SRQ-20) [70]	Women experience EA were significantly more likely to report symptoms of psychological distress
Johnson (2021) [38]	Multivariate	Material hardship	11-item index asking individuals about their ability to meet basic financial needs in the past 12 months (e.g., go hungry)	EA was positively associated with material hardship
Nicholson et al. (2018) [39]	Multivariate	Peer bullying	Four (4) items from the Panel Study of Income Dynamics Child Development Supplement III	Presence of EA at Year1 and Year 3 was associated with higher levels of peer bullying at Year 9
Postmus et al. (2012) [40]	Multivariate	Parenting engagement	Average of individual’s engagement in eight parenting activities (e.g., reading stories)	Mothers at Year 1 who experienced EA had higher odds of experiencing depression and using spanking as a form of punishment at Year 5
		Use of spanking	Frequency with which mother spanked child when they misbehaved or acted up in the past 1 month	
		Maternal depression	Composite International Diagnostic Interview Short Form (CIDI-SF) [71]	
Postmus et al. (2012b) [4]	Multivariate	Economic self-sufficiency	15-item Economic Self-Sufficiency Scale [72]	Experiencing any form of EA compared to no EA was associated with a decrease in economic self-sufficiency
Postmus et al. (2021) [41]	Multivariate	Food insecurity	One (1) item that asked participants how often people in their home go without food due to lack of money	Experiencing EA was associated with a greater likelihood of reporting food insecurity and an increase in depressive symptoms
		Depression	20-item Center for Epidemiologic Studies Depressed Mood Scale (CES-D) [59]	
Sauber et al. (2020) [42]	Multivariate	PTSD	17-item civilian version of the PTSD Checklist (PLC-C) [73]	Economic control was positively associated with PTSD and negatively associated with economic self-sufficiency; Employment sabotage was positively associated with depressive symptoms
		Depression	20-item Center for Epidemiologic Studies Depressed Mood Scale (CES-D) [59]	
		Economic self-sufficiency	15-item Economic Self-Sufficiency Scale [72]	
Stockl & Penhale (2015) [43]	Multivariate	Physical health	50-items that asked women about physical and psychological health, history of injuries, and weight problems	EA was associated with greater odds of experiencing gastrointestinal syndromes, psychosomatic symptoms, pelvic problems, allergies, and psychological problems in the past year, as well as problems to keep weight
		Mental health		
Stylianou (2018) [44]	Multivariate	Depression	20-item Center for Epidemiologic Studies Depressed Mood Scale (CES-D) [59]	EA was positively associated with depression
Tenkorang & Owusu (2019) [45]	Multivariate	Cardiovascular health	One (1) item asking if participants had ever been told by a doctor that they had diabetes, high blood pressure, high cholesterol, or stroke	Employment sabotage was positively associated with psychosocial health issues; Economic exploitation was positively associated with worse psychosocial health and greater odds of cardiovascular diseases; Economic deprivation was positively associated with worse psychosocial health and greater odds of cardiovascular diseases
		Overall health	One (1) item that asked participants to describe their health in general	
		Psychosocial health	11-items that asked participants if they had experienced a range of mental health symptoms (e.g., felt nervous)	
Usta et al. (2007) [46]	Bivariate	Common health complaints in general practice	Participants were asked to indicate how frequently they experienced complaints common in general practice (e.g., headache)	EA was positively correlated with frequency of heart palpitations and physical complaints
Voth Schrag (2015) [47]	Multivariate	Material hardship	11-item index asking individuals about their ability to meet basic financial needs in the past 12 months (e.g., go hungry)	Reporting EA was associated with a greater likelihood of depression and increased odds of experiencing material hardship
		Depression	Composite International Diagnostic Interview Short Form (CIDI-SF) [71]	
Voth Schrag et al. (2019) [48]	Multivariate	PTSD	20-item PTSD Checklist for DSM-5 (PCL-5) [74]	EA was associated with increased depression, PTSD, and economic hardship
		Depression	7-item Center for Epidemiological Studies Depression Scale Short Form (CES-D-SF) [75]	
		Economic hardship	13-item Economic Hardship Index [2]	
Voth Schrag et al. (2020) [49]	Multivariate	Economic hardship	13-item Economic Hardship Index [2]	Higher levels of EA were associated with higher levels of economic hardship
Yau et al. (2020) [50]	Multivariate	Anxiety	14-item Hospital Anxiety and Depression Scale (HADS) [76]	EA was associated with greater odds of anxiety, depression, and psychosomatic symptoms
		Depression		
		Psychosomatic symptoms	15-item Patient Health Questionnaire-15 [77]	
Yunus et al. (2017) [51]	Bivariate	Mortality	Mortality was tracked via phone calls with participants or their families followed by cross checking	Mortality was highest among individuals who experienced EA

#### Covariates

The most commonly used covariates across studies were other forms of IPV. Physical abuse was included in approximately 63% of analyses, followed by psychological/emotional abuse (49%), and sexual abuse (26%). Other covariates tended to be demographic characteristics such as age, relationship status, education level, children (either whether the respondent had children [binary] or the number of children [continuous]), and income. Race/ethnicity was included in almost every study conducted in the United States, but only in one study conducted outside of the United States (Ghana) [24]. Although used much less frequently, employment status was controlled for in 23% of studies. Only one study controlled for gender [29], as most studies included entirely female samples. Finally, seven studies included no covariates [30, 32, 45–47, 50, 51]; this was typically due to the type of analytic strategy used.

### Statistical approaches

All but six studies used regression-based analytic methods to examine the impact of economic abuse on various outcomes. Three studies used longitudinal multilevel modeling to look at the effects of economic abuse over time [3, 20, 25]. Fourteen studies used hierarchical linear regression, ordinary least squares regression, multiple regression, or Taylor Linearization to predict the association between economic abuse and a continuous outcome variable [3, 4, 20, 26, 27, 31, 34, 38–40, 42, 44, 45, 48, 49]. Thirteen studies used logistic regression to predict the odds that survivors will experience a particular outcome based on experiencing economic abuse [22–24, 28–34, 36, 37]. Other methods used included chi-square tests [39, 41, 43], t-tests [45, 46], analysis of variance [47], and correlations [50, 51].

### F indings on the impact of economic abuse

Study findings are presented in Table 2, along with information regarding how each outcome of interest was measured.

Most studies looked at financial and mental and physical health impacts of economic abuse, although some studies also examined parenting and child outcomes, and quality of life; a small number of studies included outcomes outside of these areas.

#### Financial

Economic or financial consequences of economic abuse were examined by 10 studies. Most studies found that economic abuse was associated with negative financial impacts. One longitudinal study by Adams et al. found that within-woman change in economic abuse over time was negatively associated with change in financial resources over time [3]. Five studies found that economic abuse was significantly associated with increased material [38, 47] or economic hardship [2, 48, 49]. Voth Schrag found that depression partially mediated the association between economic abuse and material hardship [47]. Further, social support moderated the relationship between economic abuse and material hardship, such that at lower levels of economic abuse, higher levels of social support were associated with fewer material hardships [49].

Some studies looked at specific economic abuse tactics. Adams et al. found that economic abuse (measured as a scale) was not significantly associated with outstanding debt but the economic exploitation subscale was [21]. Similarly, the authors also found that the economic abuse scale was not significantly associated with material dependence, but the economic restriction subscale was. Adams et al. found that coerced debt was significantly associated with greater odds of credit damage and financial dependency (meaning survivors stayed in a relationship longer because of concerns about financially supporting themselves or their children) [22]. Experiencing any form of economic abuse [4] and economic control in particular [42] were both significantly associated with lower economic self-sufficiency.

#### Mental health

While there were some discrepancies, most studies found economic abuse to be associated with various facets of mental health. Depression was the most frequently examined mental health outcome. Two longitudinal studies examining the effects of economic abuse on maternal depression over time found that experiencing economic abuse was associated with greater odds of experiencing depression [40, 47]. Seven of the cross-sectional studies found that economic abuse [27, 31, 36, 41, 44, 48, 50] and its associated tactics (i.e., employment sabotage) [24] was significantly and positively associated with depression. One study found no significant difference in depression among one-month postpartum women based on economic abuse exposure [26]. Three studies found economic abuse to be significantly and positively related to anxiety [27, 31, 36]; another two found economic abuse to be significantly positively related to PTSD [36, 42] and suicidal ideation [42, 48]. However, a study looking at an all-Latina sample of IPV survivors found that while economic abuse and depression were significantly positively correlated, economic abuse did not uniquely predict depression, anxiety, or PTSD after controlling for other forms of IPV [48]. Voth Schrag et al. found that material hardship partially mediated the relationship between economic abuse and depression, as well as economic abuse and PTSD [50].

Other components of mental health that studies looked at included self-esteem, psychosocial health, and psychological problems. Experiencing economic abuse was found to be significantly and negatively associated with self-esteem [31], psychosocial health [45], and positively associated with symptoms of psychological distress [37]. One study by Stockl and Penhale looked at the association between economic abuse and psychological problems by women’s age group [43]. Women between the ages of 66–86 had significantly greater odds of experiencing mild or strong psychological symptoms, whereas women between the ages of 16–49 had greater odds of experiencing strong psychological problems [43]. Hamdan-Mansour et al. looked at the association between economic abuse and six dimensions of psychological wellbeing. Two dimensions (self-acceptance and environmental mastery) were negatively correlated with economic abuse; the remaining four dimensions (autonomy, positive relation with other, personal growth, purpose in life) were not statistically significant [32].

Lastly, Antai et al. looked at the relationship between four economic abuse items, psychological distress, and suicide attempts [23]. An affirmative response to the item “controlled money or forced her to work” or “ever lost job/source of income because of husband” was associated with greater odds of a prior suicide attempt. An affirmative response to the items “destroyed personal property/pet or threaten to harm pet” or “ever lost job/source of income because of husband” was associated with greater odds of psychological distress. Curiously, an affirmative response to the item “disallowed respondent to engage in legitimate work” was associated with lower odds of psychological distress. Antai et al. suggested this finding could be a function of cultural norms around what is perceived as economic abuse or a function of how legitimate work is viewed (e.g., a source of psychological distress); additional research is needed to better understand this finding.

#### Physical health

Six studies looked at the association between economic abuse and physical health outcomes. One study by Stockl and Penhale looked at the association between economic abuse and several physical health outcomes by women’s age group [43]. Women between the ages of 16–49 experiencing economic abuse had greater odds of experiencing pelvic problems and difficulty keeping weight. Women between the ages of 50–65 had greater odds of experiencing psychosomatic symptoms, gastrointestinal symptoms, allergies, and difficulty keeping weight [43]. Yau et al. also found that women experiencing economic abuse had greater odds of psychosomatic symptoms [50]. Usta et al. surveyed women in health clinics about whether they were experiencing 19 common complaints in general practice and found that economic abuse was positively correlated with frequency of heart palpitations and physical complaints, although it is unclear which specific symptoms physical complaints is referring to [46]. Tenkorang and Owusu looked at physical health outcomes based on experiences with specific economic abuse tactics, specifically economic exploitation, employment sabotage, and economic deprivation [30]. Economic exploitation and economic deprivation were both significantly associated with cardiovascular disease and economic deprivation was associated with poorer perceptions of overall health; employment sabotage was associated with poorer mental health but not physical health [30]. Gurkan et al. explored the association between economic abuse and a range of pregnancy-related symptoms: gastrointestinal, reproductive, cardiovascular, mental health, neurological, dermatological, respiratory, urinary, and tiredness or fatigue [45]. Both fatigue and mental health symptom scores were significantly higher for women experiencing economic abuse [45]. Lastly, Yunus et al. looked at the associations between IPV and mortality among a sample of older adults and found that proportions of death were highest for survivors of economic abuse, although the number of mortalities in the sample was low overall [51].

#### Parenting and child outcomes

Some studies looked at associations between experiencing economic abuse and parenting behaviors and child-related outcomes. Three of these studies were longitudinal in nature and were, therefore, able to examine the impacts of economic abuse over time. However, a limitation of these analyses is that they all used the same dataset (i.e., Fragile Families). As part of the Fragile Families studies, mothers were surveyed in hospitals post-child birth (baseline) and then again when their children were ages 1, 3, 5, and 9, referred to as Y1, Y3, Y5, and Y9, respectively. Researchers found that mothers’ who experienced economic abuse in Y1 and Y3 had lower levels of parental involvement with their children and a greater likelihood of neglecting their child at Y5 [34]. Further, this economic abuse and neglect were associated with greater child delinquency in Y9; this relationship was partially mediated by parenting behaviors (i.e., physical punishment, parental involvement, child neglect). Postmus et al. found that mother’s economic abuse at Y1 and Y3 had greater odds of using spanking to discipline child at Y5, but economic abuse was not significantly associated with engagement in parent-child activities in Y5 [40]. Nicholson et al. found that economic abuse at Y1 and Y3 were also associated with higher levels of peer bullying for children in Y9; this relationship was mediated by parental involvement and this was moderated by race/ethnicity [39]. The results showed that increased parental involvement was associated with increased peer bullying for boys [39]. One cross-sectional study looked at associations between mother’s experiencing economic abuse and their perpetration of child abuse, but found that economic abuse was not significantly associated with emotional or physical child abuse perpetration [29].

#### Quality of life

While a cross-sectional study conducted by Gul et al. did not find economic abuse to be significantly associated with survivors’ contentment with life score [20], a longitudinal study by Adams and Beeble found economic abuse was significantly, negatively associated with change in the quality of life over time [25]. A second longitudinal study also looked at the association between economic abuse; economic control was initially significantly and negatively associated with quality of life, however, the relationship was no longer significant after controlling for other indicators of financial empowerment (e.g., financial knowledge, economic self-sufficiency) [29].

#### Other

Four studies examined outcomes that did not fit well in the other thematic areas previously discussed. One longitudinal study using Fragile Families data looked at the association between experiencing economic abuse in Y1, Y3, and Y5 and mother’s criminal justice involvement, defined as whether mother was charged with a crime or booked by police for anything other than a minor traffic violation in the last 4 years, at Y9; odds of experiencing criminal justice involvement were higher for mother’s experiencing economic abuse when controlling for all other forms of IPV [28]. Another longitudinal study using Fragile Families data looked at the effect that economic abuse at Y1 had on union formation at Y5; mothers experiencing economic abuse had lower odds of being married or cohabiting with baby’s father at Y5 [33]. Jewkes et al. found that economic abuse was not significantly associated with women’s discussion of HIV with their partner, however, women who suggested condom use in the past year were more likely to be financially abused [35]. Finally, Postmus et al. found that economic abuse was indirectly associated with food insecurity, as the relationship was fully mediated by depression [41].

## Discussion

The purpose of this study was to conduct a scoping review to examine the literature on IPV to better understand the effects of economic abuse on survivors. A total of 35 manuscripts met the inclusion criteria for the study. These studies examined associations between economic abuse and financial outcomes, mental and physical health impacts, parenting and child outcomes, quality of life, survivors’ criminal justice involvement, and the navigation of HIV and condom use in intimate relationships. As such, the studies had both substantive and methodological differences.

Overall, studies found significant associations between economic abuse and a range of outcomes. With regard to the methods reported within the included studies, only three studies specifically measured the effects of economic abuse on survivor outcomes longitudinally. These three studies analyzed their data using multilevel modeling to look at the effects of economic abuse over time [3, 20, 25]. Although six studies used the Fragile Families and Child Wellbeing Study dataset [28, 33, 34, 39, 40, 47], these studies used regression analyses to look at associations between experiences of economic abuse and outcomes of interest. Future research should include the use of more rigorous research methods, such as longitudinal designs, to examine the short and long-term impacts of economic abuse on survivors, as well as the directionality between relationships.

Many of the studies looked at mental health outcomes associated with economic abuse. Researchers found that economic abuse is associated with increased depression, anxiety, suicidal ideation, and PTSD. These are consistent with the mental health outcomes associated with other forms of IPV (e.g., Bonomi et al.) [78–80]. There is a need for additional research that explores a wider range of outcomes, including physical health consequences, as fewer studies examined the physical health consequences of economic abuse. Those that did generally operationalized their physical health outcomes of interest differently; therefore, it is not yet possible to draw any overarching conclusions about the impact of economic abuse on physical health, and other less-studied outcome areas. However, preliminary findings suggest that economic abuse is associated with some physical health impacts, which is also consistent with research on other forms of IPV [30, 43, 45, 46, 50, 51]. There is a need for research studies to operationalize outcomes with more consistency, so that findings can be compared across studies. Moreover, only a small number of studies looked at the indirect effects of economic abuse on survivors. Additional research is needed on factors that mediate the effects of economic abuse on various outcomes.

While all forms of IPV can impact a survivors’ economic well-being either directly or indirectly, the impact of economic abuse is particularly damaging to survivors’ economic stability. Across studies, the financial and economic impacts of economic abuse were operationalized in a range of ways. Studies included measures of economic hardship, perceptions of financial resources, debt and credit damage, and financial dependence on an intimate partner. Regardless of how these impacts were measured, all studies found statistically significant associations between economic abuse and these various facets of economic hardship. Thus far, only a handful of studies have examined the economic impacts of IPV by measuring economic abuse separately to determine whether these impacts differ from those caused by other forms of IPV (e.g., physical abuse, psychological abuse). For example, Adams et al. found that economic restriction was positively associated with material dependence on an abusive partner and outstanding debt, whereas physical abuse and psychological abuse were not [21]. However, studies often looked at the association between economic abuse and economic hardship related outcomes without controlling for other forms of IPV. While these studies make important contributions to the literature given the limited information available on economic abuse, particularly when compared to other forms of IPV, it is difficult to ascertain whether the impacts of economic abuse contribute to economic hardship above and beyond the impacts of other forms of IPV. Continued research is needed to better understand what economic abuse tactics are most harmful to survivors and interactions between other forms of IPV.

Future research should also examine economic abuse experiences and associated impacts across a broader sample. Almost all of the studies included in this scoping review examined the experiences of female survivors with male intimate partners. There is a need to understand how economic abuse manifests among other survivor samples, such as male survivors with female abusive partners and within the LGBTQIA+ community. Other scholars have stressed the need to include male victims in studies as well, including Hines et al. who found that 38% of male survivors in their study reported experiencing economic abuse [81]. While some studies looked at economic abuse among survivors later in life [43, 51], the majority focused on individuals of reproductive age. The impacts of economic abuse may vary based on survivors’ stage of life, which has been found for physical/sexual IPV and psychological abuse [82]. Similarly, economic abuse and its effects may differ based on an individuals’ socioeconomic status. Although some studies included financial circumstances (e.g., employment status) in their analyses as control variables, additional research is needed to better understand whether economic abuse and its effects differ by household income.

Some scholars have highlighted the ways in which cultural norms, including gendered attitudes around money, family dynamics, and formal and informal economic policies (e.g., unequal rights to inheritance), influence survivors’ experiences with economic abuse [83]. For example, in some cultures women may be restricted from engaging in work activities due to familial obligations, such as caring for children or elderly family members [83]. Women may also be expected to keep their financial assets in joint accounts controlled by their intimate partner, which further decreases their financial dependence [84]. Wedding-related traditions, such as marriage gifts, bride price, or dowry can also be used as forms of economic control or exploitation [85]. However, few studies considered how cultural variations may influence survivors’ experiences with and the impacts of economic abuse, as well as their help seeking behaviors. Future research should continue to explore the ways in which cultural values impact survivors’ perceptions of economic abuse and subsequently its impacts.

All of the studies included in this review used self-reported measures of economic abuse. A range of validated and non-validated economic abuse instruments were used across studies. While there was overlap across instruments, there were also substantive differences that decrease their comparability. For example, four different variations of the original Scale of Economic Abuse (SEA) were used: the original SEA [2]; the Scale of Economic Abuse-12 (SEA-12), which is an abbreviated version of the original scale [52]; the Chinese translation of the SEA-12 [50]; and the SEA2, which is a revised version of the SEA [21]. Half (17) of the articles did not use any validated measure of economic abuse. As Postmus et al. point out, it is necessary for researchers to continue to validate measures of economic abuse among diverse populations to determine whether all aspects of economic abuse are accurately represented and that the measures being used are relevant across different cultural contexts [86]. It will not be possible to collect accurate data on the prevalence and impact of economic abuse until reliable and valid measures are consistently used to assess the issue. Scholarship in this area can also elucidate whether certain forms of economic abuse are particularly harmful to survivors, both in terms of its mental and physical health impacts, as well as its financial impacts.

While most outcomes, with the exception of mental health, were measured inconsistently across studies, this is particularly true of the financial outcomes examined. Among those included across studies, economic/material hardship (two studies used an 11-item index of material hardship; three used a 13-item index of economic hardship) and economic self-sufficiency (two used the Economic Self-Sufficiency Scale) were assessed most similarly. The remaining financial outcomes (e.g., credit damage, material dependency, outstanding debt) were all measured differently, often using individual items that were not previously validated. This is not surprising, given that economic abuse research is still in its infancy [17] and fewer economic measures have been tested with IPV survivors, however, it does speak to the need for continued measurement research in this area.

Further, few studies looked holistically at the impact of economic abuse on various facets of financial wellbeing. The Consumer Financial Planning Bureau defines financial wellbeing as a state in which an individual has control over day-to-day finances, has the capacity to absorb financial shock, is on track to meet financial goals, and has the financial freedom to make choices that promote enjoyment in life [87]. However, no studies looked at the impact of economic abuse on all facets of financial wellbeing collectively. Although not discussed within the context of financial wellbeing, the Economic Self-Sufficiency Scale [72] is perhaps the measure most closely aligned with financial wellbeing that was used. The individual items in this scale represent various facets of financial wellbeing (e.g., financial freedom), although it does not adequately capture whether a survivor is on track to meet their financial goals. Kutin included measures of financial resilience (defined as the ability to absorb financial shocks) and financial stress (defined as household cash flow problems) in one study exploring risk factors for economic abuse and found limited financial resilience and moderate to high levels of financial stress were associated with greater odds of experiencing this form of IPV [86]. However, additional research is needed to better understand these bidirectional relationships, including longitudinal studies, using more comprehensive measures of financial wellbeing.

### Limitations

Although rigorous methods were used to conduct this scoping review, this study has limitations. This review focused only on quantitative studies exploring the impact of economic abuse on survivors. Future research should conduct a scoping or systematic review of the qualitative studies available and explore similarities and differences in overall study findings. Given the current state of literature in this area, the majority of studies included in this review were cross-sectional in nature. As such, directionality cannot be determined. Some studies used analytic strategies that would not allow for the inclusion of confounding factors. More rigorous, longitudinal research is needed to better understand the relationship between economic abuse and its impact over time. As noted, there were also variations in how economic abuse and outcomes of interest were measured. These variations in the operationalization of measures across studies hinders scholars’ ability to pool available data for meta-analyses [88].

Studies were limited to English-language manuscripts. While gender of the abusive partners was sometimes unclear, they appeared to be primarily male with female survivors. Further, approximately one-third of the studies recruited participants from domestic violence organizations. As such, the samples included individuals who were at higher risk for economic abuse. S tu dy findings are not representative of all survivors of IPV nor the broader population.

### Implications and future directions

This study suggests several implications and directions for future research and practice. First, although a handful of studies have examined the mediators and moderators between economic abuse and a particular outcome, the evidence is still far to understand the complex nature of economic abuse. Thus, continued research is needed to investigate how certain outcomes are produced after economic abuse, and how to protect survivors of IPV from subsequent adversity. These studies will provide critical rationales for intervention design and service implementation. Second, further studies should be conducted in diverse populations. The majority of studies concentrate on heterosexual relationships and male-to-female abuse. However, economic abuse can occur in any intimate relationship regardless of with same or opposite-sex partners. Future research should take into consideration the prevalence and consequences of economic abuse in LGBTQIA+ survivors. In addition, existing studies are primarily interested in its impacts on the survivors. However, economic abuse can impact individuals beyond direct victimization. Child development can be greatly affected when living with an economically abusive dynamic between caregivers. Thus, the continued investigation of child outcomes after economic abuse is warranted.

Third, given the body of evidence that suggests economic abuse is likely to co-occur with other forms of IPV, research should explore whether economic abuse is more harmful with the co-occurrence of other forms of IPV. In practice, service providers should be aware of the unique impacts of economic abuse and the potentially compounding effect with other forms of IPV. Domestic violence advocates should utilize comprehensive screening tools that include economic abuse to assess survivors’ IPV experiences. Domestic violence shelters and agencies should also provide quality training to workers to administer the tools appropriately and effectively. Further, given that the available evidence suggests that economic abuse can have myriad impacts on survivors, additional attention must be paid to developing and evaluating interventions that can financially empower survivors.

## Conclusion

This scoping review provides a comprehensive overview of the quantitative research focused on examining the impacts of economic abuse on survivors of IPV. Study findings highlight the wide-ranging impacts that economic abuse has on survivors globally, including their financial wellbeing and mental and physical health. However, it also illuminates gaps in the literature that provide opportunities for future research. In particular, there is a need for additional longitudinal research to explore the effects of economic abuse and other forms of IPV on survivors’ financial wellbeing over time. There is also a need for research to be conducted with broader samples of survivors, including LGBTQ+ survivors.

## Data Availability

The datasets generated and/or analyzed during the current study are not publicly available due to ongoing research but are available from the corresponding author on reasonable request.
